# Determinants of the management learning performance in ERP context

**DOI:** 10.1016/j.heliyon.2020.e03689

**Published:** 2020-04-08

**Authors:** Carlos J. Costa, Manuela Aparicio, Joao Raposo

**Affiliations:** a ISEG (Lisbon School of Economics & Management), Universidade de Lisboa, Portugal; b NOVA Information Management School (NOVA IMS), Universidade Nova de Lisboa, Portugal; c Instituto Universitario de Lisboa (ISCTE-IUL), Portugal

**Keywords:** Enterprise resource planning, ERP, Success model, Performance, Management learning, Information systems, Computer science, Human-centered computing, Education, Information science, Business

## Abstract

Management learning poses some challenges, firstly students should identify all administration areas and secondly, they should understand the big picture of an organizational context, by integrating all the studied areas. Enterprise Resource Planning (ERP) systems are the backbone of any organization, in terms of information management systems integration. The usage of these systems is important in terms of management in any organization, and ERP's can facilitate the management learning process. The main objectives of this study are to understand if the ERP usage supports management learning, and to identify the main determinants of individual performance. This study presents a success model of ERP usage for learning management context. The model was validated empirically through a survey answered by university management students. The results show that system quality, process quality, and training play a determinant role in the students' performance.

## 1 Introduction

To remain competitive on an increasingly global and technological market lead organisations to connect and converge information from various departments into a central and integrative solution (Finney and Corbett, 2007). Enterprise resource planning systems (ERP) give companies an efficient and effective integrative tool as it stores and shares business processes and information through the entire organisation in real-time (Hayes and McGilsky, 2007). Given the influence of enterprises systems on organisations performances, more and more companies are adopting this software to stay competitive and not being left behind. Thus, a market, where once only large enterprises could afford these complex and expensive systems, have been changing, due to, the demand for smaller companies for a more affordable, flexible and straightforward package. Chaudhari and Ghone (2015) predicted that in 2020 the market value would be 38.208 thousand euros, revealing the growth of 7.2% compared with 2014. The increasing adoption of ERP systems, implementation success rates are between 20% to 40% (Sun et al., 2015).

Moreover, Panorama Consulting (2017) reports that 37% of organisations in 2016, with ERP systems already implemented, received less than 50% of its expected benefits. Students' knowledge of ERP complex knowledge is a significant predictor of competency. Thus higher education adoption of ERP in their curricula increase skills of their students when providing sophisticated technology, such as ERPs. Given the importance of the ERP systems, higher education establishments have also realised its possible benefits and decided to incorporate enterprises system into their study programs (Hepner and Dickson, 2013; Maas et al., 2018). University management students understand that while using these systems, they are learning various managements concepts such as business cross-functionality, decision making, cooperation and coordination within the organisation (Aldholay et al., 2018; Boykin and Benjamin Martz, 2004; Mandal and Flosi, 2012). This empirical knowledge is considered more efficient and long-lasting and valued by companies as it guarantees better equipped and prepared employees (Chadhar and Daneshgar, 2018; Chauhan and Jaiswal, 2016). Although there are studies on ERP usage in university contexts (Binyamin et al., 2019; Rabaa'i et al., 2009; Schwald, 2011; Soliman and Karai, 2015), our question is still pertinent. The main goals of this study are to understand if the ERP usage supports management learning, and to identify the main success determinants, such as user satisfaction, use, and individual performance.

The contributions of this study are twofold. Firstly, this study indicates that ERP's usage is adequate for management learning purposes, and secondly, it presents a theoretical model of the ERP success determinants in management university learning context. In this study, we carried a quantitative methodological approach to validate the research model. We conducted a survey and 221 university management students participated voluntarily. We treated data using Partial Least Squares/Structural Equation Models' method.

The paper is composed of seven sections. The first two sections present the theoretical background in enterprise systems, state of the art about ERP usage and success. The third section contains the proposed research model. The fourth section describes the empirical methodology and the way the model was validated. The fifth and sixth sections present the study results and discussion. The final section describes the conclusions of the study.

## 2 Literature review

### 2.1 Enterprise resource planning

Enterprise resource planning (ERP) systems known nowadays come a long way since it evolved from material requirements planning (MRP) used in 1960 and 1970 and from manufacturing resource planning (MRP-II) a decade later (Chou et al., 2005; Kumar et al., 2002). Accordingly, Klaus et al. (2000), defined ERP as a set of integrated solutions that supports all the functions of an organization. As an integrative business solution, ERP shares common data manage cross-department processes workflows apply reliable business rules, and helps to implement standard proceeding through company's functional departments (Hayes and McGilsky, 2007; Kumar et al., 2002; Léger et al., 2011; Robert Jacobs & ‘Ted’ Weston, 2007; Willems and Bhuiyan, 2006). These systems assume a modular structure and all data generated throughout departments are stored in a central database and made available to others functional areas in real-time (Davenport, 1998; Elragal and Haddara, 2012; Robert Jacobs & ‘Ted’ Weston, 2007; Scott and Vessey, 2000). Traditionally enterprises systems were used by large organizations operating in intensive capital industries. However, systems development and maturation and the increasing number of suppliers, allowed small and medium enterprises, from other business areas, to purchase affordable ERP packages (Shehab et al., 2004). Enterprises adopt and implement ERP systems to obtain some benefits, such as lower costs, improve response time to customers and enhance overall performance, however, many companies fail to achieve the expected benefits due to system complexity, lack of internal expertise and user resistance (Mayeh et al., 2016). Consequently, over the last decades research in ERP increased (Costa et al., 2016) and the most investigated theme is implementation success, although system usage and evolution are considered relevant as well (Esteves and Bohórquez, 2007; Moon, 2007). However, further research in “ERP in education” topic is required, as there is more demand for students with ERP skills (Andera et al., 2008; Grabis et al., 2015). Supported in the ERP concept we collected in the central information systems' digital libraries (ACM, IEEE, Scopus) and selected papers related to ERP, adoption models, and ERP usage for education purposes, then we chose the most related papers with our primary research goal.

### 2.2 ERP empirical studies

ERP systems provide a broad research field as it is a powerful and multidisciplinary and sophisticated solution for the modern business challenge. When searching for published literature, it is evident that most of the research still focuses on ERP implementation success, however other ERP lifecycles phases, like ERP usage and benefit and ERP adoption, have earned importance in the research literature. This is due to the fact, that small-sized enterprises already have an ERP implemented and to unsuccessful ERP implementation or the vast market offer (Eden et al., 2014; Esteves and Bohórquez, 2007; Moon, 2007). Identical to organizations, universities recognize the importance of being acquainted with information technology evolution and the possible benefits of its use. Thus, the integration of information systems concepts and tools on educational curricula has a main topic for tertiary institutions (Bradford et al., 2003).

Researchers tend to use adoption theories to explain the deciding path that an individual goes through until the actual execution of the activity (Sussman and Siegal, 2003). Theory of Reasoned Action (TRA) is the main contributor of adoption theories, as it suggests that people from an intention to perform a behavior, based on their beliefs (Wallace and Sheetz, 2014).

Building on this theory, Davis (1986) presented a model that could apply Ajzen's theory to information technologies. Technology Acceptance Model (TAM) demonstrates why users adopt, or not, specific information technology to perform a job (Wallace and Sheetz, 2014). Information System researchers have widely used TAM since its conception of explaining and predicting systems use (Moon and Kim, 2001; Sternad and Bobek, 2013). When researching information system success, most authors use DeLone and McLean model. Although much of empirical research uses the above models to explain ERP life cycles on organizations, only a few research applied to the education field adopts quantitative models (Alshare and Lane, 2011; Richardson and Tan, 2005). Instead, most studies choose qualitative methods to explain the process and benefits of ERP introduction in students curricula (Beekhuyzen and Gasston, 2002; Bradford et al., 2003; Hardaway et al., 2008; Hawking et al., 2005; Schwald, 2011; Stewart et al., 2004; Strong et al., 2006; Theling and Loos, 2005).

### 2.3 Adoption models

As mention before the Theory of Reasoned Action (TRA) is the source of many information system theories. Researchers proved TRA usefulness in predicting and explaining behavior a wide variety area successfully (Davis et al., 1989). Fishbein and Ajzen (1975) point out that a person's prior intention along with their beliefs, at a given moment, could determine the individual's actual behavior. Thus TRA sees behavioral intention as the main predictor of actual individual behavior (Marangunić and Granić, 2015) and claims that two main aspects (people intention and subjective norms) influenced attitudes (Cheung and Vogel, 2013). The most used model systems adoption, TAM, adopts TRA central values to explain and predict person behavior when interacting with information systems. TAM explains why individuals choose to adopt or not adopt technology when performing a task (Wallace and Sheetz, 2014). Davis et al. (1989) explain the main determinants of technologies adoption through the user's behavior intention and attitude.

### 2.4 IS success model

Although users system acceptance is a necessary precondition for IS success, this factor is not equivalent to the success of IS (Petter et al., 2008). Thus, DeLone and McLean (1992) developed a model that could deal with the complexity, interdependency and multi-dimensionality nature of IS and capable of evaluating IS implementation success. The identified success dimensions are user satisfaction, intention to use, use, individual and organizational impacts. Later, individual and organizational impacts formed another success dimension, net benefits (Delone and McLean, 2003). In the DeLone and McLean model (2016) identifies six interdependent dimensions of IS success: Information Quality; System Quality; Service Quality Intention to Use/Use; User Satisfaction; Net Impacts. Net impacts were renamed by these authors, as they referred that net impacts can be benefits or not.

## 3 Model proposal

### 3.1 ERP system use and its impacts on users

**Figure 1:**
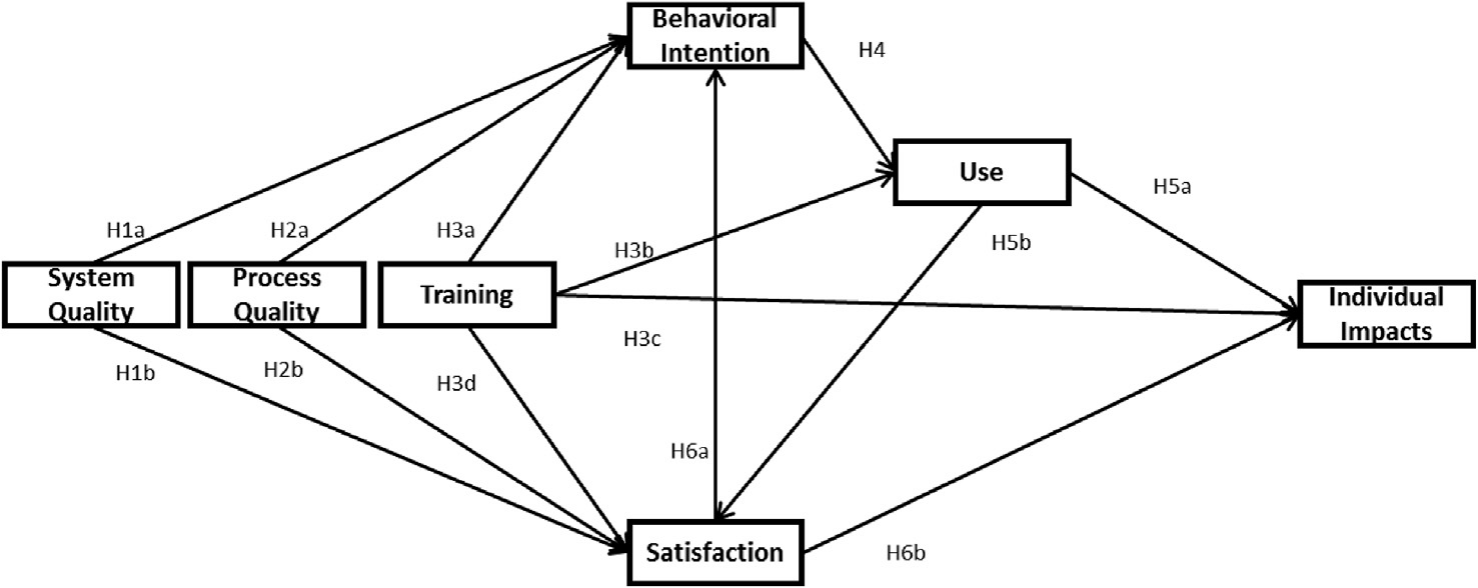
ERP research Model for management learning context.

After a revisiting the main models to study IS success and its adaptations to particular cases, a theoretical model is presented to evaluate the influence of System Quality, Process Quality and Training on Individual Impact through the effect of Behavioral Intention, Use, user Satisfaction, and individual impacts of the learners. Accordingly, and based on the IS success theory, we propose a research model for ERP usage in management learning context (Figure 1).

### 3.2 Model constructs

Costa et al. (2016) identified three critical external dimensions: system quality, process quality and Training. Supported in the literature, we also introduced three dimensions: behavioral intention (BI) and use as adoption measures and user satisfaction and individual impacts to assess system success. System quality is the degree to which a system is easy to use or has the desired functional characteristics to effortlessly accomplish user's tasks (Delone and McLean, 2003; Schaupp et al., 2006). Empirical researches show that system quality has a significant influence on IS success as it displayed the higher score, therefore being considered one of the most important external factors (Petter and McLean, 2009; Urbach et al., 2010). Even though some authors say, that system quality already includes the process quality dimension (Urbach et al., 2010) believes that these two dimensions have a different meaning. Besides the ability to support business processes, Process Quality also concerns the level of required ERP's customization to address adequately and efficiently those business processes (Urbach et al., 2010). As a result of learning paradigm change, from a theoretical view to a more “hands-on-material” and ERP complexity, training is considered a critical factor. Rajan and Baral (2015) showed that training has a significant influence on how users perceived systems easiness (Amoako-Gyampah and Salam, 2004; Yusuf et al., 2004). Thus, the training dimension corresponds to the skills of users for tasks accomplishment (Ruivo et al., 2014, p. 170). Behavioral intention construct refers to an individual's subjective probability of performing a specified behavior (Davis et al., 1989), and is deeply related to the actual use of the system. Much of the empirical research proved that the higher intention to use the system most likely will actual system use (Petter and McLean, 2009; Rajan and Baral, 2015). Use corresponds to the practical usage of the system in the job context (Davis, 1986). Almost every researcher, when studying IS success, adopts measures to evaluate user satisfaction and the impacts on users. System's user satisfaction describes user's feelings after interacting with the system.

User satisfaction is the most popular success measure to assess IS success (DeLone and McLean, 2016). Furthermore, empirical research showed a significant influence on user satisfaction on individual impact (Iivari, 2005; Petter and McLean, 2009; Urbach et al., 2010). This last dimension is responsible for measuring the effect of using an enterprise system on users behavior and performance (DeLone and McLean, 1992; Junior et al., 2019).

### 3.3 Model hypothesis

Delone and McLean (2003) introduce intention to use to their model when pinpointing the multidimensional features of use, hence establish a relationship between system quality and Intention to use. Davis et al. (1989), when trying to bring together the best of TRA and TAM model, studied the relationship between perceived ease of use (most used measured of System Quality) and behavioral intention. Further studies have analyzed this relation using Information Success model (Chien and Tsaur, 2007; Mardiana et al., 2015; Petter and McLean, 2009), Technology Acceptance Model (Davis, 1986; Rajan and Baral, 2015; Sternad and Bobek, 2013; Venkatesh and Davis, 2000, 1996), hybrid models (Costa et al., 2016) and found a positive effect between these dimensions. Trough the IS success model, DeLone and McLean (1992) stated that system quality affects user satisfaction. As the system quality is perceived to be higher by users, the more satisfied they are with the ERP (Iivari, 2005). Several researchers have proven a positive impact of System Quality on User Satisfaction (Chien and Tsaur, 2007; Costa et al., 2016; Petter and McLean, 2009; Urbach et al., 2010). Thus, we hypothesize:

H1a ERP System Quality has a positive effect on students Behavioral Intention.

H1b ERP system Quality has a positive effect on Students Satisfaction.

According to Urbach et al. (2010), as referred on literature review, process quality is distinct from system quality, therefore should be considered as another IS quality measure. Empirical research conducted by Urbach et al. (2010) found a not significant but positive effect from Process Quality on Use. Although Urbach et al. (2010) choose not to study the effect on behavioral intention, due to specific characteristics of their research, the authors apply and follow information system success model (DeLone and McLean, 1992), where the intention to use and use are gathered under the same construct. This allows for predicting a possible relationship between user satisfaction and behavioral intention. The level of customization is a critical factor when implementing an ERP. Although the process's customization may lead to higher costs, it can help users better-understanding business processes and address its problems and needs. Research on process quality effects on user satisfaction led to conclude that there is positive, but small influence (Urbach et al., 2010). Thus, we theorize:

H2a ERP Process Quality have a positive effect on students Behavioral Intention.

H2b ERP Process Quality have a positive effect on students Satisfaction.

Training promotes interaction with ERP systems leading to a favorable attitude towards the system and erasing negative perceptions (Rajan and Baral, 2015), reducing training, and its costs may result in user's negative attitudes against the system (Lassila and Brancheau, 1999). When provided as part of ERP process implementation, training will affect users beliefs about system benefits (Amoako-Gyampah and Salam, 2004). Teaching how to use the system will improve familiarity and boost its use (Ruivo et al., 2014). Empirical research from Ruivo et al. (2014) proved this statement to be accurate, demonstrating on their research that training has positive and significate relation with the usage of the system (use). Allocation of the necessary training resources will increase user satisfaction (Bradford and Florin, 2003). The research done by Bradford and Florin (2003) indicated a positive relationship between training and user satisfaction. Training and education will reduce employees' anxiety and stress about the use of the ERP system and provide a better understanding about the benefits of the system for their tasks (Lee et al., 2010). Training increases the users' confidence in their ability to use them. Hence, we assume:

H3a Training students on ERP have a positive effect on students Behavioral Intention.

H3b Training students on the ERP have a positive effect on students Use of the system.

H3c Training students on ERP have a positive effect on students Satisfaction.

H3d Training students on ERP have a positive effect on Individual Impacts.

Fishbein and Ajzen (1975) sustain that the actual behavior of a user is determined by their behavioral intention to perform the referred behavior. Accordingly, to research done by Venkatesh and Davis (1996) user's Behavioral Intention is a better predictor of system use than other metrics used. Extensive empirical research also points out positive and many cases, the strong relationship between these two dimensions (Davis, 1989; Sternad and Bobek, 2013; Venkatesh and Davis, 1996, 2000). Hence, we expect:

H4 Students Behavioral Intention has a positive effect on students' Use of the system.

DeLone and McLean (1992), when presenting their information system success model, settled that use has a direct effect on individual impacts. Several studies support this relationship, by finding a positive and significant association between system use and individual impacts (Aldholay et al., 2018; Almutairi and Subramanian, 2005; Kositanurit et al., 2006; Petter and McLean, 2009). Furthermore use is also interrelated with users satisfaction not only in a process sense but as well in a casual sense, where a positive experience with system use will lead to higher user satisfaction (Delone and McLean, 2003). Other studies (Chiu et al., 2007; Petter and McLean, 2009) found a positive and significant effect of use in user satisfaction. Thus, as the previous authors, we expect:

H5a ERP use has a positive effect on Individual Impact

H5b ERP use has a positive effect on students Satisfaction

User satisfaction is an antecedent of behavior intention in a casual sense as user's satisfaction will dictate their intention to use the system (Delone and McLean, 2003). Studies investigating the relationship between user satisfaction and use have found moderate support for this relationship (Iivari, 2005). Urbach et al. (2010) found a small but positive effect on Behavioral intention by user satisfaction. User satisfaction is direct antecedent and predictor of individual impacts (DeLone and McLean, 1992). A research review, from several empirical studies, by Petter et al. (2008) indicates a strong support of user satisfaction positive effect on individual impact. Thus, we can assume:

H6a Students Satisfaction with ERP have a positive effect on their Behavioral Intention.

H6b Students Satisfaction have a positive effect on Individual Impact.

## 4 Empirical methodology

### 4.1 Measurement instrument

After the selection of suitable constructs from the literature review, quantitative methods validated the research model. Each construct contains several measurement items developed and tested by previous empirical research. Nevertheless, each model's construct and measurement item were carefully discussed and selected and adapted, considering the model's validity and characteristics of the study. To empirically test the model, firstly, a brief introduction to the ERP system was lectured to students, afterwards, was handed a walk-trough exercise, providing the hands-on-task opportunity. Lastly, students answered a survey where they evaluated the model (see Table 1).

**Table 1:** Model dimensions.

Constructs	Definition	Authors
System Quality	The degree to which a system is easy to use or has the desired functional characteristics to accomplish user's tasks effortlessly	(Delone and McLean, 2003; Schaupp et al., 2006)
Process Quality	Required level of customization to adequately and efficiently support an organization's business processes	(Urbach et al., 2010),
Training	A measure of user training easiness, implying clear insights into the system contents, and to browse through the system to perform daily tasks.	(Amoako-Gyampah and Salam, 2004; Ruivo et al., 2014; Yusuf et al., 2004)
Behavioral Intention	The subjective probability that an individual has a specified behavior.	(Davis, 1989)
Use	individual's actual direct usage of the given system in the context of his or her job	(Davis, 1986)
User Satisfaction	The effective attitude of a user after system interaction.	(DeLone and McLean, 1992; Urbach et al., 2010)
Individual Impact	The effect of ERP on user behavior and performance.	(DeLone and McLean, 1992)

### 4.2 Data collection

The empirical study was directed to university management students with backgrounds in administration and technologies with different levels of knowledge. The questionnaire was created supported by validated scales (Appendix A), which was delivered through an online platform. Thus, it was selected different classes from bachelors and master's degrees to provide a broader range of answers and increase the model's quality. The study was applied in courses with the permission of teachers, where the students had one hour and a half to complete a proposed exercise and then respond to a questionnaire. The process of data collection followed a strict ethical path, upon which, after research approval the university, we presented the questionnaire to students, we informed all respondents about the academic research purpose of the survey, all the respondants participated voluntarily, and the involved university did not oppose the study. All the participants answered this questionnaire, and 57% of the respondents were end-users at work in several companies, they used this type of systems in their routine organizational tasks. This exercise leads students to experiment and visualize cross-functionality of business processes and apply previous knowledge lectured in theoretical courses. The survey was sent electronically, via an e-learning platform to students. The study was carried during the academic year 2016/2017, summing a total 229 answered questionnaires, however only 221 were considered valid due to incomplete questionnaires. Table 2 displays the sample characteristics.

**Table 2:** Sample characteristics.

Sample Characteristics		n = 221
**Gender**		
Male	74	33%

Female	147	67%

**Instruction level completed**		

High School (undergraduate students)	93	42%
Bachelor	76	34%
Postgraduate	14	6%
Master	38	17%

## 5 Analysis and results

### 5.1 Assessment of measurement model

In order to assess the variables relationships, it was used the structural equation model (SEM) with the partial least square. PLS is used to validate the causality of structural models, theoretically explained previously. We use this approach to study the relations between system quality, process quality, training (independent variables) and behavioral intention, use, user satisfaction, individual impacts (dependent variable). PLS is adequate for this research study as we can use it in small samples with non-normal distribution. Moreover, it decreases the residual variance of the dependent variables (Hair et al., 2011). Although the selected constructs had already been used by previous research, the measurement model was tested to evaluate the constructs' reliability and validity. Hence, the measurement model was examined through the usage of different tests, such as construct reliability, internal reliability, convergent validity and discriminant validity. To verify the construct reliability, we employed the composite reliability measure rule that 0.800 can be considered reliable (Coelho and Henseler, 2012). In Table 3, every constructs' results are above 0.900.

**Table 3:** Measurement model results.

Construct	Item	Loading	Internal reliability	Composite reliability	Cronbach's Alpha	AVE	Discriminant Validity
System Quality (SysQ)	SysQ1	0.837	0.700	0.923	0.901	0.668	Yes
	SysQ2	0.849	0.720				
	SysQ3	0.831	0.690				
	SysQ4	0.854	0.730				
	SysQ5	0.726	0.528				
	SysQ6	0.801	0.642				
Process Quality (ProcQ)	ProcQ1	0.808	0.653	0.934	0.918	0.669	Yes
	ProcQ2	0.823	0.677				
	ProcQ3	0.841	0.707				
	ProcQ4	0.817	0.668				
	ProcQ5	0.817	0.667				
	ProcQ6	0.811	0.658				
	ProcQ7	0.808	0.653				
Trainnig (Train)	Train1	0.902	0.813	0.937	0.899	0.832	Yes
	Train2	0.922	0.850				
	Train3	0.912	0.831				
Behavioral Intention (BI)	BI1	0.968	0.938	0.968	0.935	0.939	Yes
	BI2	0.970	0.940				
Use	Use1	0.950	0.903	0.941	0.876	0.889	Yes
	Use2	0.936	0.876				
User Satisfaction (Sat)	Sat1	0.873	0.762	0.951	0.931	0.828	Yes
	Sat2	0.921	0.848				
	Sat3	0.934	0.873				
	Sat4	0.910	0.829				
Individual Impacts (II)	II1	0.893	0.797	0.963	0.954	0.813	Yes
	II2	0.918	0.843				
	II3	0.918	0.843				
	II4	0.931	0.867				
	II5	0.887	0.786				
	II6	0.863	0.745				

Furthermore, construct Cronbach's alpha demonstrated equal reliability for all items (Hair et al., 2011). For convergent validity measuring, each construct average variance extracted (AVE) was assessed. AVE is a measure that allows evaluating if a construct can capture at least half of the item's variance, therefore demonstrate convergence (Coelho and Henseler, 2012; Fornell and Larcker, 1981). From AVE results (Table 3), all constructs are above 0.600, concluding the existence of a high constructs' convergence validity.

Finally, to evaluate the construct's discriminant validity, two tests were conducted. First, indicators loadings should be higher than the cross-loadings (Hair et al., 2011). In appendix B, all indicators loadings (in bold) are higher than the cross-loadings. The second test is a more conservative approach and uses Fornell-Larcker criterion, which validates the constructs' discriminant by comparing the square root of AVE with the results of inter-construct correlation (Fornell and Larcker, 1981). The main principle of this criterion is that each construct shares more variance with its items than with other construct's items. Table 4 exhibits AVE square root (in bold diagonal) are higher than, and the results of inter-construct correlation also evidencing constructs' discriminant validity. In conclusion, the measurement model exceeded every test, proving its reliability and validity after this confirmation is possible to assess the structural model using PLS (Hair et al., 2011).

**Table 4:** Interconstruct correlation and the square root of AVEs.

	SysQ	ProcQ	Train	BI	Use	Sat	II
SysQ	**0.816**						
ProcQ	0.551	**0.818**					
Train	0.495	0.491	**0.912**				
BI	0.449	0.337	0.431	**0.969**			
Use	0.225	0.083	0.354	0.451	**0.943**		
Sat	0.531	0.568	0.520	0.400	0.342	**0.910**	
II	0.461	0.501	0.467	0.474	0.272	0.571	**0.902**

Interconstruct correlation coefficients and square root of AVE (in bold on diagonal).

### 5.2 Assessment of structural model

**Figure 2:**
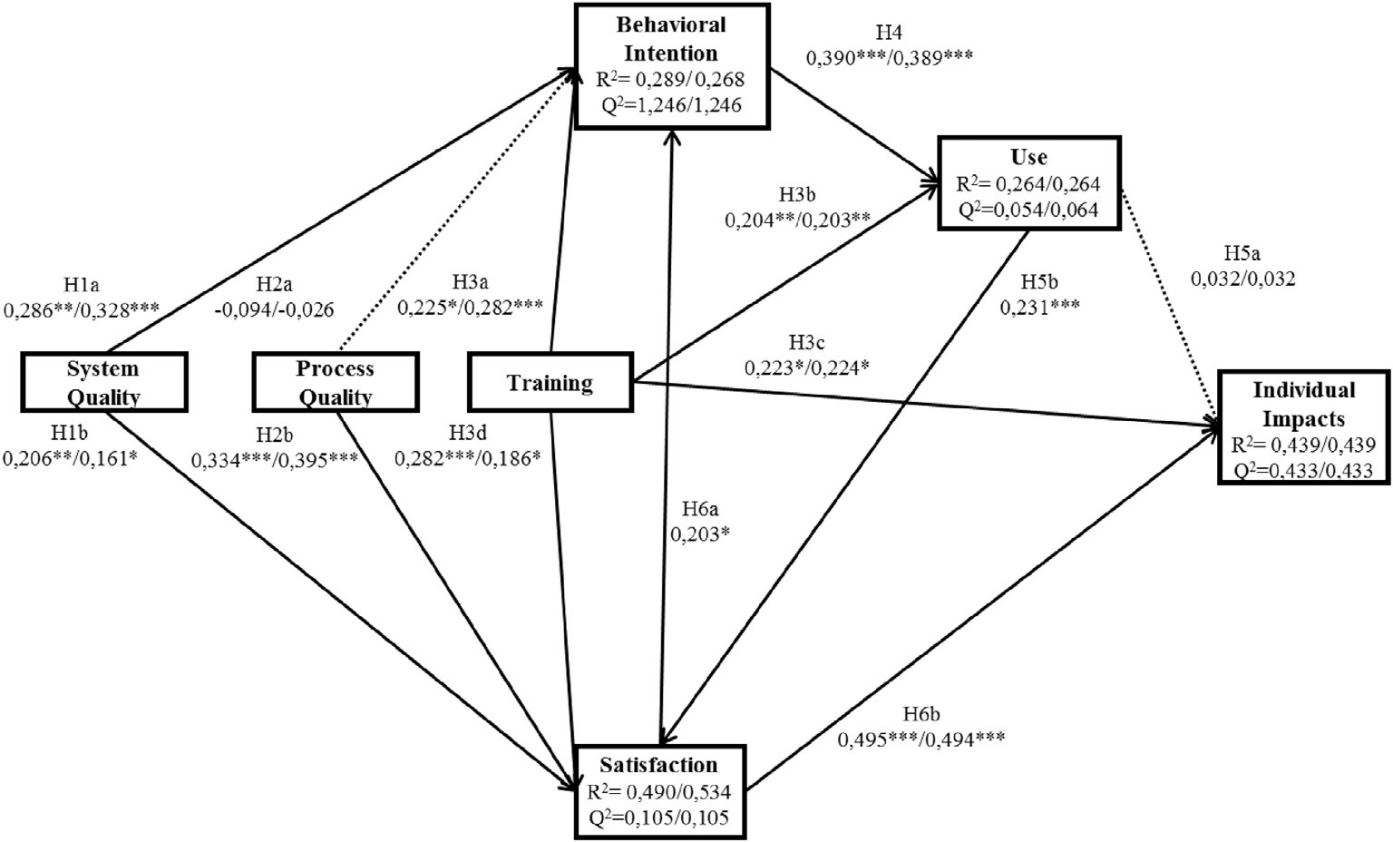
Structural model results.

The quality of the structural model was then evaluated through bootstrapping and PLS algorithm. Bootstrapping is a resampling technique that draws many subsamples retrieved from the original dataset. For this research, 5000 subsamples were used to determine the path's significance within the structural model. These two previous tests had to be made twice, as hypotheses H5b and H6a when evaluated at the same time create a loop making impossible to measure a single model, thus it was required to assess two models. Model 1 measures the impact of user's satisfaction on the user's behavioral intention (H6a), whereas model 2 undertakes the influence of system users into user's satisfaction (H5b). Figure 2 shows the results of the structural model.

The model explains 28.9%/26.8% (model 1/model 2) of the variation in behavioral intention. System quality significantly explains behavioral intention (β = 0.286 and ρ < 0,010/β = 0.328 and ρ < 0.001), confirming H1a. Process quality does not show statistically significant support behavioral intention (ρ = 0.256/ρ = 0.752), leading to H2a rejection. Training significantly explains behavioral intention (β = 0.225 and ρ < 0.050/β = 0.282 and ρ < 0.001), approving H3a. User satisfaction demonstrates significantly support to behavioral intention (β = 0.203 and ρ < 0.050), therefore confirming H6a. User satisfaction variation is explained in 49%/53.4% (model 1/model 2) by the structural model. System quality significantly explains student's satisfaction (β = 0.206 and ρ < 0.010/β = 0.0161 and ρ < 0.050). Process quality also shows to be statistically significant explaining of user satisfaction (β = 0.334 and ρ < 0,001/β = 0.395 and ρ < 0.001). Follow the same conclusion, training can significantly explain user satisfaction as well (β = 0.282 and ρ < 0.001/β = 0.186 and ρ < 0.050). Also, use significantly explains user satisfaction (β = 0.231 and ρ < 0.001). As the above paths were appraised as statistically significant, this means that H1b, H2b, H3d, H6b are all supported. System actual usage is significantly explained by behavioral intention (β = 0.390 and ρ < 0.001/β = 0.389 and ρ < 0.001) and training (β = 0.204 and ρ < 0.010/β = 0.203 and ρ < 0.010), moreover these two constructs explain 26% of the variation in system use. Thus, we may support H4 and H3b. The structural model explains 43.9% of the variation in student's impacts. Training significantly explains individual's impacts (β = 0.223 and ρ < 0.050/β = 0.224 and ρ < 0.050), confirming H3b. User satisfaction also shows to be statistically significant explaining individual impact (β = 0.495 and ρ < 0.001/β = 0.494 and ρ < 0.001) therefore supporting H6b. Contrary to these paths, use is not statistically significant explaining individual impact (ρ = 0.581/ρ = 0.582). Thus, H5a is not supported. Structural model's quality is assessed trough squared multiple correlations (R^2^) and model's predictive accuracy (Q^2^). After the validation of these two measures and assessment of the model's quality, it was concluded that the model is valid. The latent variable's coefficient path was analyzed to study hypotheses and if it had predictive evidence, and it was considered significant (ρ < 0.050) the relationships between constructs were considered supported. Table 5 summarizes all the hypotheses results.

**Table 5:** Hypotheses results.

	Independent variable		Dependent variable	Findings (Model 1/Model 2)	Effect size f^2^ (Model 1/Model 2)	Conclusion
H1a	System Quality	→	Behavioral Intention	Positively & statistically significant ∗∗/∗∗∗(β = 0.286/0.328; ρ < 0.010/ρ < 0.001)	Small/Small	Supported
H1b	System Quality	→	User Satisfaction	Positively & statistically significant ∗∗/∗(β = 0.206/0.0161; ρ < 0,010/ρ < 0.050)	Small/Small	Supported
H2a	Process Quality	→	Behavioral Intention	Negatively & not statistically significant		Not supported
H2b	Process Quality	→	User Satisfaction	Positively & statistically significant ∗∗∗/∗∗∗(β = 0,334/0.395; ρ < 0.001)	Small/Medium	Supported
H3a	Training	→	Behavioral Intention	Positively & statistically significant ∗/∗∗∗(β = 0.225/0.282; ρ < 0.050/ρ < 0.001)	Small/Small	Supported
H3b	Training	→	Use	Positively & statistically significant ∗∗/∗∗(β = 0.204/0.203; ρ < 0.010)	Small/Small	Supported
H3c	Training	→	Individual Impacts	Positively & statistically significant ∗/∗(β = 0.223/0.224; ρ < 0.050)	Small/Small	Supported
H3d	Training	→	User Satisfaction	Positively & statistically significant ∗∗∗/∗(β = 0.282/0.186; ρ < 0.001/ρ < 0.050)	Small/Small	Supported
H4	Behavioral Intention	→	Use	Positively & statistically significant ∗∗∗/∗∗∗(β = 0.390/0.389; ρ < 0.001)	Medium/Medium	Supported
H5a	Use	→	Individual Impacts	Positive & not statistically significant		Not supported
H5b	Use	→	User Satisfaction	Positively & statistically significant .--/∗∗∗ (β = .---/0.231; ρ < 0.001)	.--/Small	Supported
H6a	User Satisfaction	→	Behavioral Intention	Positively & statistically significant ∗/--(β = 0.203/.--; ρ < 0.050)	Medium/.--	Supported
H6b	User Satisfaction	→	Individual Impacts	Positively & statistically significant ∗∗∗/∗∗∗(β = 0.495/0.494; ρ < 0.001)	Medium medium	Supported

## 6 Discussion

### 6.1 Hypotheses discussion

As seen in Table 5, most of the hypotheses are empirically supported for students use of ERP systems, only H2a and H5a are not supported. Although the model's predictive ability to support all hypotheses, results demonstrate different levels of supportive effects. An analyze to inner hypotheses results (H4, H5b and H6a) shows that they have a different level of effects and strengths (significance). Hypotheses H4 and H6b demonstrate the same medium explanatory effect (0.150 > f^2^ ≤ 0.350). However, the levels of significance are different, whereas H4 demonstrates a very significant level of influence (p < 0.001), H6a only presents a low significance (p < 0.050).

The relation of system use impacts shows a positive but small effect (0.150 > f^2^ > 0.020) on user's satisfaction (H5b), nevertheless it reveals a high level of significance (p < 0.001). Roca et al. (2006) express that system managers should improve ERP attributes, as users are more willing to the system (intention) when they have trust in it (user satisfaction). In the same line, Chen (2011) on his empirical study, outlines that students are more likely to accept and use e-learning systems when they provide good performance evidence and more learning tools.

In the present research, ERP system quality showed a positive, but small, effect explaining both student's behavioral intention and satisfaction (0.150 > f^2^ > 0.020). These findings are surprising when compared with Petter and McLean (2009) results, since, postulates that system quality influences user's intention and satisfaction. Besides this level of the effect, our findings support the above statements and other studies (Costa et al., 2016; Mohammadi, 2015; Motaghian et al., 2013).

According to structural model results, process quality was found to have either small or medium positive effect on student's satisfaction depending on the model (model 1: small effect; model 2: medium effect). This change in effect classification is due to, the values proximity to range boundary of 0.150 (model 1: f^2^ = 0.110; model 2: f^2^ = 0.161). This finding meets (Urbach et al., 2010) results and could suggest that students appreciate ERP system capabilities to support effectively and efficiently the requested tasks.

In hypothesis H2a, process quality does not demonstrate evidence of supporting behavioral intention (H2a), indicating that the ERP level of customization and task support does not influence user intention to use the system.

Model results show that training does not have a significant influence on the other dimensions, displaying a small explanatory effect (0.150 > f^2^ > 0.020) on student's intention, impacts, satisfaction and system use (H3a, H3b, H3d). This small effect of training contradicts some research, as they give this construct great importance when adopting new technology (Rajan and Baral, 2015; Ruivo et al., 2014; Youngberg et al., 2009). These contradictory results may come from the level of course depth, suggesting that training should be more extensive. When addressing individual impacts, it is clear that student's satisfaction, with the ERP system, plays a significant role in their learning performance. This is supported by the results (H6b), which show satisfaction having a medium effect explaining system impacts on students (0.150 > f2 ≤ 0.350). This finding was expected, as they are in line with earlier research (Aparicio et al., 2017; Iivari, 2005; Petter and McLean, 2009; Urbach et al., 2010).

Training (H3c) is another dimension that shows to have a positive but small influence on individual impact (0.150 > f2 > 0.020). Although this positive result, previous studies put training as a crucial factor in information system success (Rajan and Baral, 2015; Ruivo et al., 2014). Bradley (2008) concluded that companies which attribute higher importance to training quantity and quality are the ones that achieved success in implementing ERP systems. Thus, it is expected that the development of an extensive and more fitting course will increase training effect on individual's impact.

Contrary to the above hypothesis, H5a was not supported, due to its level of significance (p ≥ 0.050), furthermore use construct was found to not having almost any effect on individual's impact (f2 = 0.002). This hypothesis outcomes are contractive with almost every empirical research as they not only support but also indicate a substantial influence of actual use in individual impacts (Chen, 2010). A possible explanation to this result could be the context, as a student do not have access to ERP systems more difficult is to them to experience its potential impacts.

From this study can be withdrawn that student's satisfaction towards the system, is the main factor influencing ERP benefits gaining. Thus, it is possible to assume that students value more practical learning, where they have access to everyday business tools and processes and come closer to market needs. Another important finding is the student's high system use intention, concluding that they acknowledge ERP's importance and benefits on leaning performance and in the business world and intend to use this kind of systems in class or their future jobs.

### 6.2 Theoretical implications

The present research provides a different approach to evaluate the impacts of ERP system universities curriculum. This study follows a quantitative model, which indicates the possible impacts on university students through enterprise information systems usage. Thus, this model undertakes a mix of constructs withdrawn from adoption theory (Davis et al., 1989; Venkatesh and Davis, 2000) and IS success theory (DeLone and McLean, 1992; Delone and McLean, 2003) and other research extensions (Ruivo et al., 2014; Urbach et al., 2010) creating a unique model.

This study indicates that the ERP characteristics, system quality, process quality (in terms of its adequacy to support tasks), as well as the perception of the in-depth training contribute to user satisfaction, as it also determines the intention to use, and ERP practical usage. Other important implication of this study is that training and user satisfaction influences individual impact (performance).

### 6.3 Practical implications

The proposed study provides to universities a tool capable of evaluating the impact of enterprises systems usage on student's learning. As the research above displayed, assessing ERP impacts on students is a multidimensional interdependent analysis, where some construct's relations are stronger than others.

As practical implications we derive from this study, that university lecturers need to adequate their teaching topics with the practical usage of ERP in management teaching. When students perceive that the ERP has quality, and it supports companies' business processes, this leads to students' satisfaction, thus positively influencing their management topics understanding.

### 6.4 Limitations and future work

Although the research showed good results and its model was validated, some limitations can be pointed. First, the students had a short period of training with the system. Also, the study was only conducted in one university, not representing a larger sample. Therefore, in the future need of development of more in-depth training, using various software representing the market.

The students' reaction suggests the use of gamification techniques in future classes. This may also be a subject of research. It also would be essential to conduct research studies, during management learning, such as simulating a market composed of several companies in one class, thus providing the effect on learning companies businesses' interconnections, for example using "ERPsim" (HEC, 2020). This kind of classes provides learning insights to students using effective ERP.

## 7 Conclusions

Enterprise systems are considered a very useful tool for business management, is almost a pre-requisite for modern and competitive organizations. As an increasing number of companies adopt and implement these information systems, the demand for employees with ERP skills increase. Despite this market need, few are the universities using a practical approach to complement ERP education. Besides the opportunity of addressing the market gap and give students a better employment rate, universities should look to ERP software as a teaching tool considering it enables a better linkage and understanding of management concepts.

This empirical research developed a theoretical model to find the success determinants of ERP system used in the education field. It was hypothesized, considering information system's literature review, that the main factors influencing impacts on students were system quality, process quality and training. The influence of these dimensions was channelled through other relevant constructs: behavioral intention; actual use; satisfaction; individual impacts. All these model dimensions were validated, therefore legitimizing the proposed model. The research was tested on university students from different backgrounds and degree levels, gathering a total of 221 questionnaires answers. The collected data allowed the validation of the measurement and structural model's results. Research's results showed that from the three independent variables used, system quality and training have a positive influence on student's intention to use the system.

Regarding student's satisfaction, all them (system quality, process quality and training) were found to a positive impact on it. Another finding demonstrates that satisfaction has a positive effect on behavioral intention concluding that more satisfied students have a more significant disposition to use the system in the future. Satisfaction was also found to be the critical determinant success factor when assessing the impacts of using enterprises systems on education, whereas actual usage did not show evidence of having a positive influence on individual impacts, it displayed a positive influence on user's satisfaction. Although training showed a positive effect on student's impacts, this effect was classified as small, falling short to literature review expectations. These findings lead to the conclusion that enterprise information systems success in education may be driven from a positive student's satisfaction towards the system.

## Declarations

### Author contribution statement

Carlos J. Costa, Manuela Aparicio: Conceived and designed the experiments; Analyzed and interpreted the data; Contributed reagents, materials, analysis tools or data; Wrote the paper.

Joao Raposo: Performed the experiments; Wrote the paper.

### Funding statement

This research did not receive any specific grant from funding agencies in the public, commercial, or not-for-profit sectors.

### Competing interest statement

The authors declare no conflict of interest.

### Additional information

No additional information is available for this paper.

## Appendix A Supplementary data

Suplementary_Material_Appendix_A Suplementary_Material_Appendix_A

Suplementary_Material_Appendix_B Suplementary_Material_Appendix_B
