# Supplementary material for: Determinants of the management learning performance in ERP context
Source: Heliyon. 2020 Apr 8;6(4):e03689. doi: 10.1016/j.heliyon.2020.e03689 (PMC7150519; doi:10.1016/j.heliyon.2020.e03689)
Supplement: Suplementary_Material_Appendix_A [file mmc1.docx]

**Supplementary Material: Appendix A**

# Appendix A: Cross Loadings

| **Construct** | **Questionnaire Items** | **Author** |
| --- | --- | --- |
| System Quality | SysQ1:Our ERP is easy to navigate.  SysQ2:Our ERP allows me to easily find the information I am looking for.  SysQ3:Our ERP is well structured.  SysQ4:Our ERP is easy to use.  SysQ5:Our ERP offers appropriate functionality.  SysQ6:Our ERP offers comfortable access to all the business applications I need. | (Venkatesh et al., 2012) |
| Process Quality | ProcQ1:Our ERP software supports the work processes efficiently.  ProcQ2:Our ERP software supports the work processes reliably.  ProcQ3:Our ERP software supports the work processes accurately.  ProcQ4:Our ERP software supports the work processes easy initiation  ProcQ5:Our ERP software supports the work processes in a way that allows one to understand them.  ProcQ6:Our ERP software supports the work processes in a way that allows one to trace them.  ProcQ7:Our ERP software supports the work processes fully. | (Urbach et al., 2010) |
| Behavioral Intention | BI1:I intend to continue using the ERP in the future.  BI2:I will always try to use the ERP in my daily life.  BI3:I plan to continue to use the ERP frequently. | (Venkatesh et al., 2012) |
| ERP usage | Use1: At present, I consider myself to be a frequent user of the ERP.  Use2:I currently use the ERP | (Davis et al., 1992) |
| User Satisfaction | Sat1:The ERP supports adequately my area of work and responsibility  Sat2:The ERP is efficient  Sat3:The ERP is effective  Sat4:The ERP satisfies me on the whole | (Urbach et al., 2010) |
| Individual Impact | II1:The ERP enables me to accomplish tasks more quickly  II2:The ERP improves my job performance  II3:The ERP increases my productivity  II4:The ERP enhances my job effectiveness  II5:The ERP makes it easier to accomplish tasks  II6:The ERP is useful for my job | (Urbach et al., 2010) |
| Training | Train1:The training course on the system was very in-depth  Train2:The content course training was very understandable  Train3:Navigating through the topic formats of the training resources was very coherent to daily tasks | (Ruivo et al., 2014) |
| Intention to Use (Behavioral Intention) | BI1:Assuming I have access to the system, I intend to use it.  BI2:Given that I have access to the system, I predict that I would use it. | (Venkatesh et al., 2012) |
