# Supplementary material for: Determinants of the management learning performance in ERP context
Source: Heliyon. 2020 Apr 8;6(4):e03689. doi: 10.1016/j.heliyon.2020.e03689 (PMC7150519; doi:10.1016/j.heliyon.2020.e03689)
Supplement: Suplementary_Material_Appendix_B [file mmc2.docx]

**Supplementary Material: Appendix B**

# Appendix B: Cross Loadings

|  | **SysQ** | **ProcQ** | **Train** | **BI** | **Use** | **Sat** | **II** |
| --- | --- | --- | --- | --- | --- | --- | --- |
| **SysQ1** | **0.837** | 0.490 | 0.437 | 0.434 | 0.283 | 0.531 | 0.462 |
| **SysQ2** | **0.849** | 0.583 | 0.444 | 0.467 | 0.307 | 0.488 | 0.480 |
| **SysQ3** | **0.831** | 0.613 | 0.449 | 0.269 | 0.100 | 0.475 | 0.417 |
| **SysQ4** | **0.854** | 0.596 | 0.478 | 0.428 | 0.227 | 0.473 | 0.401 |
| **SysQ5** | **0.726** | 0.479 | 0.410 | 0.254 | 0.036 | 0.406 | 0.346 |
| **SysQ6** | **0.801** | 0.555 | 0.434 | 0.369 | 0.159 | 0.477 | 0.384 |
| **ProcQ1** | 0.567 | **0.808** | 0.424 | 0.255 | 0.043 | 0.477 | 0.452 |
| **ProcQ2** | 0.565 | **0.823** | 0.367 | 0.184 | 0.009 | 0.497 | 0.410 |
| **ProcQ3** | 0.532 | **0.841** | 0.445 | 0.241 | 0.056 | 0.529 | 0.475 |
| **ProcQ4** | 0.551 | **0.817** | 0.475 | 0.368 | 0.100 | 0.503 | 0.468 |
| **ProcQ5** | 0.567 | **0.817** | 0.431 | 0.401 | 0.121 | 0.542 | 0.506 |
| **ProcQ6** | 0.479 | **0.811** | 0.418 | 0.241 | 0.113 | 0.477 | 0.410 |
| **ProcQ7** | 0.595 | **0.808** | 0.504 | 0.259 | 0.061 | 0.539 | 0.452 |
| **Train1** | 0.515 | 0.489 | **0.902** | 0.370 | 0.335 | 0.506 | 0.419 |
| **Train2** | 0.535 | 0.523 | **0.922** | 0.384 | 0.283 | 0.538 | 0.468 |
| **Train3** | 0.435 | 0.462 | **0.912** | 0.456 | 0.407 | 0.521 | 0.523 |
| **IntBI1** | 0.454 | 0.371 | 0.419 | **0.968** | 0.459 | 0.438 | 0.513 |
| **IntBI2** | 0.443 | 0.302 | 0.443 | **0.970** | 0.472 | 0.416 | 0.506 |
| **Use1** | 0.248 | 0.110 | 0.387 | 0.484 | **0.950** | 0.356 | 0.289 |
| **Use2** | 0.202 | 0.056 | 0.321 | 0.419 | **0.936** | 0.353 | 0.280 |
| **Sat1** | 0.496 | 0.502 | 0.489 | 0.459 | 0.418 | **0.873** | 0.556 |
| **Sat2** | 0.537 | 0.597 | 0.527 | 0.344 | 0.285 | **0.921** | 0.546 |
| **Sat3** | 0.541 | 0.600 | 0.485 | 0.380 | 0.312 | **0.934** | 0.567 |
| **Sat4** | 0.548 | 0.572 | 0.577 | 0.418 | 0.352 | **0.910** | 0.634 |
| **II1** | 0.436 | 0.511 | 0.455 | 0.481 | 0.288 | 0.601 | **0.893** |
| **II2** | 0.498 | 0.548 | 0.485 | 0.436 | 0.257 | 0.621 | **0.918** |
| **II3** | 0.447 | 0.462 | 0.436 | 0.430 | 0.271 | 0.538 | **0.918** |
| **II4** | 0.477 | 0.527 | 0.493 | 0.497 | 0.258 | 0.551 | **0.931** |
| **II5** | 0.469 | 0.507 | 0.439 | 0.474 | 0.215 | 0.555 | **0.887** |
| **II6** | 0.437 | 0.455 | 0.492 | 0.525 | 0.342 | 0.559 | **0.863** |
